# Identification of Candidate Cotton Genes Associated With Fiber Length Through Quantitative Trait Loci Mapping and RNA-Sequencing Using a Chromosome Segment Substitution Line

**DOI:** 10.3389/fpls.2021.796722

**Published:** 2021-12-14

**Authors:** Quanwei Lu, Xianghui Xiao, Juwu Gong, Pengtao Li, Yan Zhao, Jiajia Feng, Renhai Peng, Yuzhen Shi, Youlu Yuan

**Affiliations:** ^1^School of Biotechnology and Food Engineering, Anyang Institute of Technology, Anyang, China; ^2^State Key Laboratory of Cotton Biology, Institute of Cotton Research, Chinese Academy of Agricultural Sciences, Anyang, China

**Keywords:** QTL mapping, RNA-seq, CSSLs, fiber length, candidate genes

## Abstract

Fiber length is an important determinant of fiber quality, and it is a quantitative multi-genic trait. Identifying genes associated with fiber length is of great importance for efforts to improve fiber quality in the context of cotton breeding. Integrating transcriptomic information and details regarding candidate gene regions can aid in candidate gene identification. In the present study, the CCRI45 line and a chromosome segment substitution line (CSSL) with a significantly higher fiber length (MBI7747) were utilized to establish F_2_ and F_2:3_ populations. Using a high-density genetic map published previously, six quantitative trait loci (QTLs) associated with fiber length and two QTLs associated with fiber strength were identified on four chromosomes. Within these QTLs, *qFL-A07-1*, *qFL-A12-2*, *qFL-A12-5*, and *qFL-D02-1* were identified in two or three environments and confirmed by a meta-analysis. By integrating transcriptomic data from the two parental lines and through qPCR analyses, four genes associated with these QTLs including Cellulose synthase-like protein D3 (*CSLD3*, *GH_A12G2259* for *qFL-A12-2*), expansin-A1 (*EXPA1*, *GH_A12G1972* for *qFL-A12-5*), plasmodesmata callose-binding protein 3 (*PDCB3*, *GH_A12G2014* for *qFL-A12-5*), and Polygalacturonase (*At1g48100*, *GH_D02G0616* for *qFL-D02-1*) were identified as promising candidate genes associated with fiber length. Overall, these results offer a robust foundation for further studies regarding the molecular basis for fiber length and for efforts to improve cotton fiber quality.

## Introduction

Cotton (*Gossypium* spp.) is an important cash crop and the most commonly planted form of renewable textile fiber in the world. There are 52 known cotton species (Li et al., 2014, 2015), including the tetraploid lines *G. hirsutum* (Upland cotton), which exhibits high fiber yields, medium fiber strength and length, and excellent adaptability, and the *G. barbadense* lines (Sea Island, Egyptian, or Pima cotton), which exhibit lower fiber yields and reduced adaptability, but the fiber tends to be longer and stronger (Liu et al., 2016; Lu et al., 2017; Shi et al., 2019). Introgressing novel genetic material associated with fiber length and strength from *G. barbadense* into *G. hirsutum* may thus offer valuable opportunities to improve Upland cotton fiber quality.

Many quantitative trait loci (QTLs) associated with fiber quality and yields have been identified through the crossing of *G. hirsutum* and *G. barbadense* (Said et al., 2015; Abdullaev et al., 2017), with most such interspecific mapping having been performed using F_2_ (Jiang et al., 1998; Kohel et al., 2001; Paterson et al., 2003; Mei et al., 2004; Lin et al., 2005), F_2:3_ (He et al., 2012), BC_1_, BC_2_, and BC_2_S_1_ populations (Shi et al., 2020). However, accurately identifying individual QTLs in this context is challenging, given the complex genetic backgrounds in these studies and the potential interactions between QTLs and genetic backgrounds or different QTLs (Islam et al., 2016a,b).

Chromosome segment substitution lines (CSSLs) are genetic populations that carry one or a limited number of donor chromosomal segments on a recurrent genotypic background. The donor segments are the only difference between the CSSL and the recurrent parental lines. As such, CSSLs can reduce the potential genetic interference inherent in interspecific crossing-based studies, making them valuable for QTL identification and for studies of gene function, genetic effects, and gene pyramiding (Zhao et al., 2012). CSSLs have been successfully employed for studies of rice and tomato plants (Illa-Berenguer et al., 2015), and recent reports have also demonstrated their promise in the context of cotton breeding analyses (Wang et al., 2008; Yang and Li, 2009; Peng et al., 2012).

Fine-mapping is integral to the identification of candidate genes present within QTL regions, but general fine-mapping strategies tend to be time- and labor-intensive as they necessitate the development of large populations consisting of tens of thousands of members in order to accurately localize genetic variants and to screen for reliable molecular markers necessary to identify polymorphic DNA markers associated with a specific trait of interest (Zhou et al., 2013; Chang et al., 2016; Liu et al., 2016). Fiber RNA-sequencing (RNA-seq) can offer direct insight into the transcriptional regulation of a given fiber texture, making it suitable for identifying candidate genes related to cotton fiber development (X.-B. and Li et al., 2005; Machado et al., 2009; Deng et al., 2011; Shan et al., 2014; Tang et al., 2014a,b). Li et al. previously employed a normalized cDNA library from a *G. barbadense* 3–79 fiber line to identify the α-expansin family protein *GbEXPATR*. Such overexpression was associated with decreased micronaire values together with increases in fiber strength (3.8–5.8%) and length (5.9–7.7%) as compared to wild-type (WT) plants (Li et al., 2016). While QTL mapping experiments can identify genomic regions in which candidate genes may be located, RNA-seq approaches can offer direct transcriptomic insight into the expression of these genes during different stages of plant development. By integrating these two techniques, it may be possible to more efficiently identify relevant candidate genes related to cotton fiber quality (Islam et al., 2016b; Liu et al., 2016, 2019; Man et al., 2016; Lu et al., 2017; Wang et al., 2020). Liu et al. (2016) further identified three fiber quality-related candidate genes through a combination of fine-mapping and RNA-seq analyses, while Man et al. (2016) employed a microarray-mediated comparative transcriptomic approach to analyze 10 DPA fibers, leading to the identification of 212 differentially expressed genes in genomic regions corresponding to 24 yield QTLs and 11 yield trait QTL hotspots. Islam et al. (2016b) identified three QTL regions associated with the control of fiber quality in the MD52ne cotton line and speculated that receptor-like kinase pathway genes may be candidate regulators of fiber length and strength.

Based on fiber quality, we have identified multiple CSSLs within a population of 332 CSSLs derived from the Hai1 line, a conventional cultivar of *G. barbadense* with a donor parent with high fiber quality, and the recurrent parental line CCRI45, a widely grown upland cotton cultivar with a high yield bred by the Institute of Cotton Research (ICR), the Chinese Academy of Agricultural Sciences (CAAS) Anyang, Henan Province (Shi et al., 2020). The MBI7747 line, which exhibited significantly increased fiber strength and length when grown in Anyang, China (2010, 2011), and in Aksu, Xinjiang Uyghur Autonomous Region (Lu et al., 2017), was chosen from these identified CSSLs for further study.

To identify the Hai1-derived genes that may be responsible for the fiber phenotypes observed in the MBI7747 line, we herein crossed the MBI7747 and CCRI45 lines and evaluated fiber quality data in the resultant F_2_ and F_2:3_ populations, revealing QTLs associated with fiber quality. We additionally analyzed the transcriptomic profiles of fibers at 10 days post-anthesis (DPA) to identify genes that were differentially expressed in these two parental lines. Key candidate genes associated with fiber quality were then identified through the integration of these QTL and differentially expressed gene (DEG) datasets.

## Materials and Methods

### Plant Materials

In our previous analysis, we evaluated fiber quality traits for 332 CSSLs and the two associated parental lines in multiple environments (Shi et al., 2020). Based on these results, we found that the MBI7747 line exhibited significantly higher fiber length, strength, and micronaire relative to the parental line. To identify the introgressed chromosomal segment and the QTLs/genes that may account for the observed improvements in overall fiber quality in the MBI7747 line, we crossed the CCRI45 with MBI7747 lines in the summer of 2012 in Anyang, Henan, China. F_2_ seeds were obtained via the self-pollination of F_1_ plants in the winter of 2012 in Sanya, Hainan, China. In total, 604 F_2_ plants and associated parental strains were grown in the summer of 2013 in Anyang, from which 153 and 82 F_2:3_ families were chosen at random. These F_2:3_ families and CCRI45 plants were then grown in Aksu, Xinjiang, China and Anyang, Henan, China. Bolls that had opened naturally were harvested from individual parental and F_1_/F_2_/F_2:3_ plants to gin the fiber therein. Fiber samples from all collected F2 bolls and from 30 bolls per F_2:3_ family were collected and mixed for fiber testing. A high-volume instrument (HVI) system was used to assess fiber quality at the Supervision Inspection and Testing Cotton Quality Center, Anyang, China. Collected data included fiber elongation (FE, %), fiber upper half mean length (FL, mm), fiber micronaire (FM) readings, fiber strength (FS, cN/tex), and the fiber length uniformity ratio (FU, %).

### Simple Sequence Repeat Genotyping and Quantitative Trait Loci Mapping

Samples of gDNA were isolated from fresh leaves derived from parental plants and from 604 F_2_ plants via the cetyltrimethylammonium bromide (CTAB) approach. A genetic linkage map published previously (Shi et al., 2015) was used for reference, with the 2,292 simple sequence repeat (SSR) sequences therein being utilized to screen for introgressed *G. barbadense* chromosomal regions and polymorphisms when comparing CCRI45 and MBI7747 lines. Those SSRs exhibiting clear polymorphisms were utilized to genotype all 604 F_2_ plants, with PCR amplification and associated analyses being performed as detailed previously (Sun et al., 2011), after which PCR products were separated electrophoretically for microsatellite marker assessment and PAGE/silver staining, with clearly polymorphic DNA bands from these gels then being utilized for genotyping and scoring purposes. QTLs associated with fiber length and strength were identified using the QTL IciMapping v 4.0 program, with a QTL being defined based upon a log of odds ratio (LOD) ≥ 3.0 threshold. Those QTLs identified in two or three environments were considered to be stable QTLs. QTL naming consisted of a “q” followed by an abbreviation of the relevant trait, chromosome number, and serial number. For example, *qFL-A12-2* corresponds to the 2nd QTL on chromosome A12 associated with the regulation of fiber length.

### Quantitative Trait Loci Physical Location Identification

The CottonGen database^1^ was used to obtain marker information, after which candidate QTL intervals were identified based upon the sequences of flanking markers in the nearest marker, with physical flanking marker locations being determined with BLAST via the alignment of marker sequences and the *G. hirsutum* reference genome (Hu et al., 2019).

### Quantitative Trait Loci-Cluster Analysis

A QTL Meta-analysis of fiber quality analysis was performed using the BioMercator 4.2 software (Arcade et al., 2004). QTLs were projected onto the genetic map and QTL cluster analyses were performed for all traits. We used four models and each had an Akaike information criterion (AIC) value that was selected and used to identify the position of QTL clusters. QTL cluster nomenclature was as follows: Clu + the English abbreviation of trait + the serial number of the chromosome + the serial number of the cluster on the chromosome associated with the same trait (Li et al., 2019).

### Identification of Differentially Expressed Fiber Length-Related Genes in Quantitative Trait Loci Regions

Gene expression levels in MBI7747 and CRI45 lines were compared via an RNA-seq analysis of 10 DPA fibers deposited in the NCBI database (accession number: SRP084203). Cleaned sequences were mapped to the most recent *G. hirsutum* reference genome (Hu et al., 2019) using TopHat v2^2^ (Kim et al., 2013), allowing for individual mismatches but discarding instances in which multiple mismatches were evident. Those genes that were differentially expressed between MBI7744 and CCRI45 lines at 10 DPA were identified with the generalized fold change algorithm [GFOLD^3^ (Feng et al., 2012)]. GFOLD values ≥ 0.5 or ≤ −0.5 were considered indicative of DEGs that were significantly upregulated and downregulated, respectively. DEGs in fiber length QTL regions were identified based on gene names from transcriptomic data.

### qRT-PCR

Total RNA was extracted from fibers from the two parents at 5, 7, 10, 15, 20, and 25 DPA, after which qRT-PCR analyses of the five DEGs of interest associated with specific QTL regions were conducted using a 7500 Fast Real-Time PCR Instrument (Applied Biosystems, CA, United States). All primers were designed with the primer-BLAST tool^4^, and the specificity of these sequences was confirmed using BLAST^5^ (Altschul et al., 1997). Primers (Supplementary Table 1) were synthesized by Sangon Biotech Co., Ltd. (Shanghai, China). All analyses were repeated in triplicate, and the *G. hirsutum histone 3* gene (forward primer: 5′-GGTGGTGTGAAGAAGCCTCAT-3′; reverse primer: 5′-AATTTCACGAACAAGCCTCTGGAA-3′) was utilized as a normalization control. The 2^–ΔΔ*Ct*^ method was used to calculate relative gene expression.

## Results

### Analysis of Fiber Quality Phenotypes in the F_2_ and F_2:3_ Populations

Mean fiber quality trait values in MBI7747 and CCRI45 lines are compiled in Table 1. Relative to CCRI45 line, MBI7747 line exhibited longer fibers (>32 mm) that were stronger (>34 cN⋅tex-1) and had lower micronaire values, consistent with the improved fiber quality of the MBI7747 line. Fiber length and strength values for the F_2_ and F_2:3_ populations exhibited continuous variance, with average values for both of these variables being higher than in the CCRI45 line. An ANOVA revealed significant variability in the FL and FS traits in the F_2_ and two F_2:3_ populations (Table 2). Correlation analyses for FL and FS in the F_2_ and F_2:3_ populations are shown in Table 3. The F_2_ population was significantly correlated (*r* > 0.649, *P* = 0.01) with FL values for two F_2:3_ groups, consistent with a robust correlation between QTLs/genes and FL variation. The F_2_ population was also significantly correlated (*r* = 0.251, *P* = 0.05) with a single group with respect to FS (2014 kora), although this correlation was weaker than correlations observed for FL.

**TABLE 1:** Fiber quality traits for the CCRI45 and MBI7747 lines.

Sample	Environment	Fiber length (mm)	Micronaire	Fiber strength (cN/tex)	Fiber elongation (%)	Fiber uniformity (%)
MBI7747	2011AY	34.84	3.5	35.76	5.6	82.9
	2011XJ	34.19	4.1	33.20	5.8	83.2
	2012AY	32.32	3.9	35.00	6.5	86.7
	2013AY	33.92	4.0	34.50	5.9	85.1
CCRI45	2011AY	29.15	4.1	29.24	6.2	83.0
	2011XJ	28.85	4.7	27.87	6.0	83.7
	2012AY	28.93	4.2	28.78	6.5	84.3
	2013AY	29.68	4.9	29.85	6.2	85.5

**TABLE 2:** Descriptive statistics corresponding to fiber quality-related traits in the primary study populations.

Trait	Group	Year	Population	Mean	Min	Max	Range	CV (%)	Skewness	Kurtosis
FL (mm)	F_2_	2013	604	31.38	26.42	36.72	10.30	5.00	0.04	0.15
	F_2:3_ (AY)	2014	82	33.11	30.54	36.06	5.52	4.10	0.09	−0.50
	F_2:3_ (Korla)	2014	153	30.92	26.35	35.09	8.74	5.30	0.08	−0.37
FS cN/tex	F_2_	2013	604	30.88	26.50	35.70	9.20	5.00	0.05	−0.01
	F_2:3_ (AY)	2014	82	31.27	27.44	34.50	7.06	5.34	0.05	−0.58
	F_2:3_ (Korla)	2014	153	28.43	24.79	35.77	10.98	6.46	0.75	1.13

**TABLE 3:** Correlation coefficients corresponding to five fiber quality traits in the primary study populations.

Traits		FL			FS		
		F_2_	F_2: 3_ (AY)	F_2:3_ (Korla)	F_2_	F_2: 3_ (AY)	F_2:3_ (Korla)
FL	F_2_	1					
	F_2: 3_ (AY)	0.729**	1				
	F_2:3_ (Korla)	0.649**		1			
FS	F_2_	0.498**			1		
	F_2:3_ (AY)		−0.002		0.26	1	
	F_2:3_ (Korla)			0.032	0.251*		1

*At the 0.05 level highly significant correlation; **at the 0.01 level significant correlation.

### Simple Sequence Repeat Marker-Based F_2_ Population Genotyping

**FIGURE 1:**
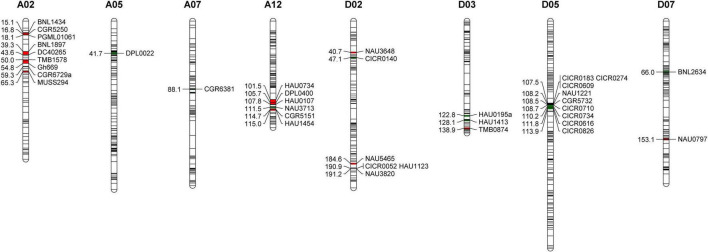
The Gossypium barbadense chromosomal segments carried by the MBI7747 line. Red represents heterozygous fragments and green represents homozygous fragments.

Of the 2,292 analyzed SSR markers, 37 pairs were found to be polymorphic between the MBI7747 and CCRI45 lines, with the GGT2.0 software subsequently being used to identify the introgressed Hai1 chromosome segments within the MBI7747 line genome (Figure 1). The MBI7747 line background recovery rate was 98.90%, with 21 *G. barbadense* introgression segments being distributed across nine chromosomes with a total coverage distance of 55.3 cM. Of these, nine homozygous fragments were 13.4 cM and 12 heterozygous fragments were 4.19 cM, accounting for 0.73 and 2.36%, respectively. More introgressive segments were observed on chromosomes A02 and D02 relative to other chromosomes, and all 37 SSR markers were utilized for the genotyping of the 604 F_2_ plants.

### Fiber Quality-Related Quantitative Trait Loci Mapping in the F_2_ and F_2:3_ Populations

**FIGURE 2:**
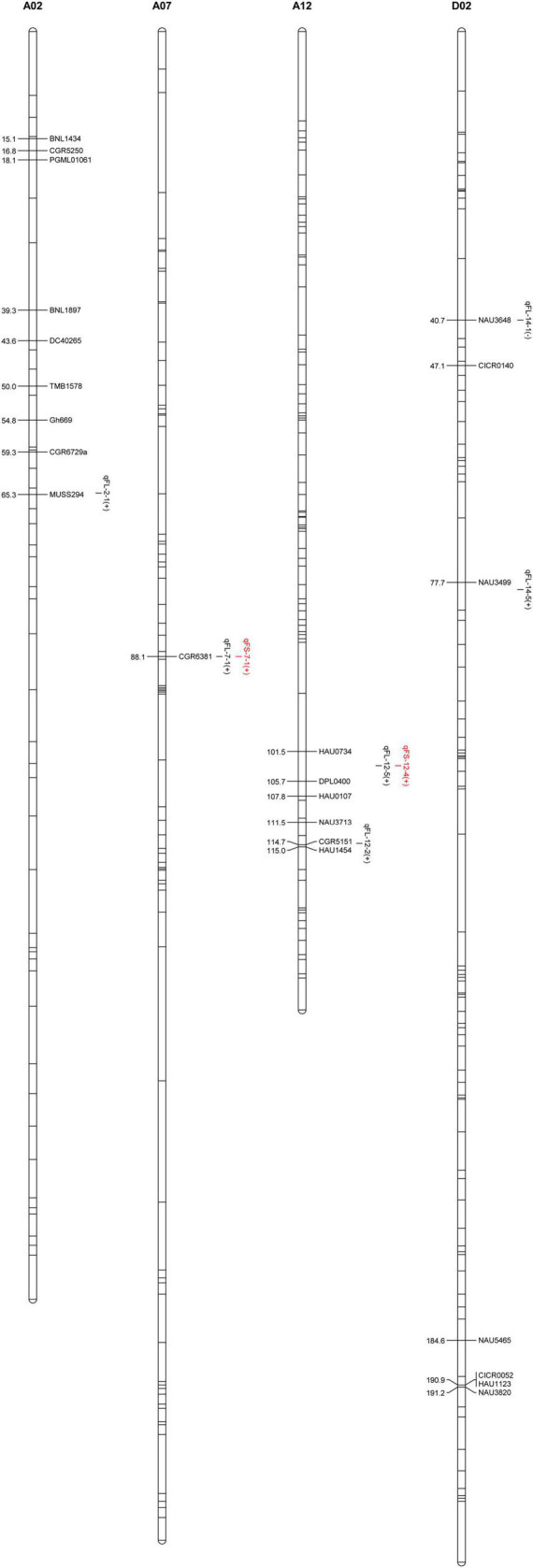
The chromosomal distribution of QTLs associated with FL and FS.

Using a previously constructed genetic map (Shi et al., 2015) and phenotypic and genotypic data from the F_2_ and F_2:3_ populations, eight QTLs corresponding to FS and FL traits were identified on chromosomes A02, A07, A12, and D02 (Table 4 and Figure 2). These included six FL-related QTLs that explained 2.05–20.99% of the observed phenotypic variability in fiber length. Five of these QTLs exhibited additive effects from 0.45 to 1.14 mm consistent with increases in FL mediated by Hai1-derived alleles, while the *qFL-D02-1* QTL exhibited additive effects from −0.2 to −0.28 mm consistent with a decrease in FL mediated by Hai1-derived alleles. Four of these FL-related QTLs (*qFL-A07-1*, *qFL-A12-2*, *qFL-A12-5*, and *qFL-D02-1*) were stable QTLs identified in two or three environments (Table 4). In addition, two FS-related QTLs were identified that, respectively, explained 3.24 and 7.73% of the observed FS phenotypic variability, with additive effects from 0.37 to 0.56 cN⋅tex-1 consistent with increases in FS mediated by Hai1-derived alleles. The *qFL-A12-5* and *qFS-A12-5* alleles mapped to the same position, suggesting that they may be tightly linked or that this QTL may be associated with pleiotropic effects. The physical distance interval of the QTLs was obtained through aligning the flanking markers sequence to the *G. hirsutum* genome (Table 4).

**TABLE 4:** Fiber quality-related QTLs detected via QTL Icimapping.

Trait	QTL	Position	Pop	Nearest marker	LOD	PVE (%)	Add	Physical distance interval
FL	*qFL-A02-1*	65.12	F2	MUSS294	6.22	4.33	0.45	A02 30835379-42214908
	*qFL-A07-1*	88.09	F2	CGR6381	9.15	6.23	0.5	A07 16434080-16741843
		88.09	F2:3XJ	CGR6381	3.17	6.28	0.6	
	*qFL-A12-2*	114.52	F2	CGR5151	12.6	10.25	0.74	A12 94389921-97556034
		114.52	F2:3XJ	CGR5151	9.29	20.99	1.14	
		114.52	F2:3AY	CGR5151	4.69	18.58	1.08	
	*qFL-A12-5*	103.52	F2	HAU0734	10.01	9.66	0.55	A12 85232423-91725336
		103.52	F2:3XJ	HAU0734	3.17	11.25	0.62	
	*qFL-D02-1*	40.73	F2	NAU3648	3.07	2.05	–0.28	D02 7266663-10929925
		40.73	F2:3XJ	NAU3648	2.51	4.45	–0.2	
	*qFL-D02-5*	78.73	F2:3XJ	CICR0140	2.61	19.74	0.96	D02 7296347-9972197
FS	*qFS-A07-1*	88.09	F2	CGR6381	9.84	7.73	0.56	A07 16434080-16741843
	*qFS-A12-5*	103.52	F2	HAU0734	3.49	3.24	0.37	A12 85232423-91725336

### Quantitative Trait Loci Meta-Analysis of Fiber Quality

**FIGURE 3:**
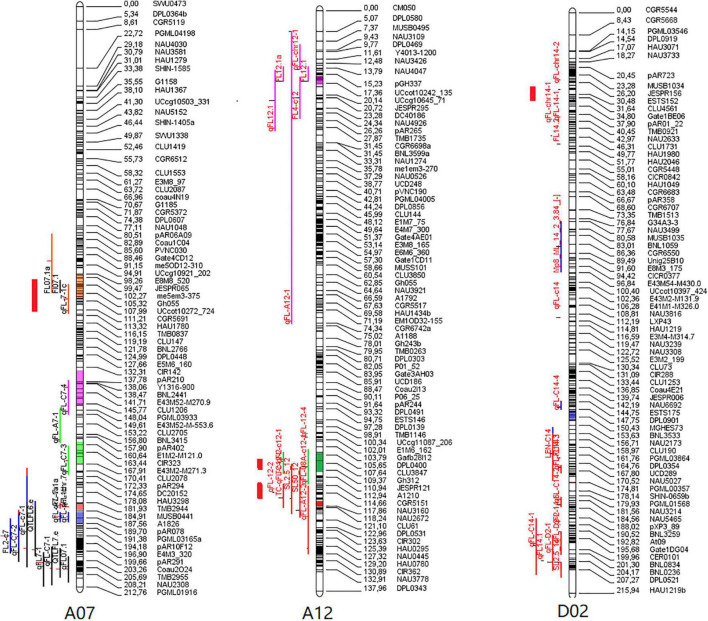
Meta-analysis of QTLs associated with fiber length on chromosomes 7, 12, and 14. The thick red lines represent the four major QTL in this study.

To better understand whether the QTLs identified above are consistent with those identified in prior studies of *G. hirsutum* × *G. barbadense* populations and to further validate our own findings, we co-localized these fiber quality-related QTLs with 1,974 QTLs from the CottonQTLdb (Said et al., 2015) and 770 other QTLs (Li et al., 2019) not included in this study database, revealing 15 QTLs on chromosomes A07, A12, and D02 to be localized within seven QTL hot spots. Four stable FL-related QTLs (*qFL-A07-1*, *qFL-A12-2*, *qFL-A12-5*, and *qFL-D02-1*) were all present within reported hot spots (Figure 3). These findings suggest these four stable QTLs to be commonly associated with FL in different genetic populations.

### Identification of FL Quantitative Trait Loci-Related Differentially Expressed Genes in RNA-Seq Data

To identify fiber length-related genes, fiber samples collected at 10 DPA from CCRI45 and MBI7747 lines were utilized for an RNA-seq analysis (Lu et al., 2017; Li et al., 2021). Following the filtering of low-quality reads, 73.4 and 73.5 million clean reads were, respectively, obtained for the MBI7747 and CCRI45 lines. In total, 3,611 DEGs (2,150 upregulated, 1,461 downregulated in MBI7747) were identified when comparing MBI7747 and CCRI45. Physical distance intervals corresponding to the four stable FL-related QTLs (*qFL-A07-1*, *qFL-A12-2*, q*FL-A12-5*, and *qFL-D02-1*) were chosen in an effort to identify FL-related genes. Based on the TM-1 genomic position, these four stable QTLs contained 793 genes (the *qFL-A07-1* contains seven genes, the *qFL-A12-2* contains 286 genes, the *qFL-A12-5* contains 312 genes, the *qFL-D02-1* contains 188 genes) of which 462 were expressed in at least one parental sample (Supplementary Table 2). However, only 59 genes (36 upregulated and 23 downregulated) within these four QTL regions were significantly differentially expressed between the two parental lines (Supplementary Table 3).

### Identification of Candidate Genes Associated With Stable FL-Related Q

**FIGURE 4:**
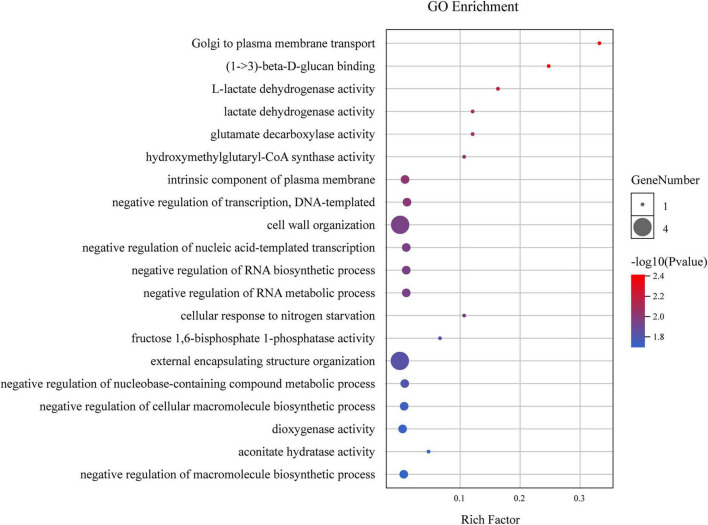
GO analysis of DEGs within the four stable QTL regions.

The 59 identified DEGs within the four stable QTL regions were primarily associated with transport from the Golgi to the plasma membrane, (1- > 3)-beta-D-glucan binding, and other GO terms. Of these terms, those most closely associated with fiber development included cell wall organization and external encapsulating structure organization (Figure 4). In the 59 identified DEGs, only four genes (*GH_A12G1972*, *GH_A12G2014*, *GH_A12G2259*, and *GH_D02G0616*) were associated with these two GO terms, and these genes were thus selected as promising candidate genes for subsequent qPCR-based analyses of their expression during different stages of fiber elongation during development.

**FIGURE 5:**
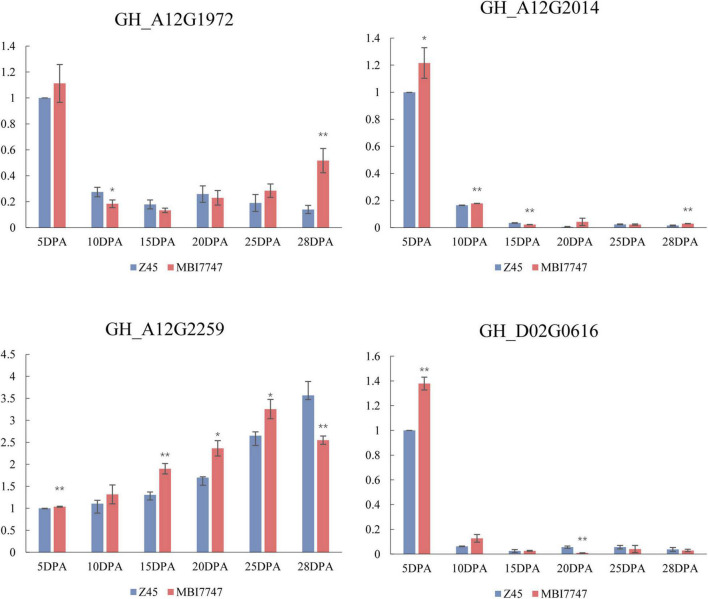
qPCR analysis of transcript levels for four candidate genes over the course of fiber elongation in the two parental lines. Relative expression levels are shown on the *Y*-axis, while different DPA values over the course of fiber elongation are noted on the *X*-axis. Error bars correspond to standard error. *At the 0.05 level highly significant correlation; **at the 0.01 level significant correlation.

The *Cellulose synthase-like protein D3* (*CSLD3*) gene (*GH_A12G2259*) associated with *qFL-A12-2* was expressed at higher levels in developing fibers from MBI7747 line as compared to those from CCRI45 plants. The *expansin-A1* (*EXPA1*) gene associated with *qFL-A12-5* (*GH_A12G1972*) was expressed at lower levels during critical fiber cell elongation periods in MBI7747 plants relative to levels in CCRI45 line. *GH_A12G2014*, encoding the plasmodesmata callose-binding protein 3 (*PDCB3*), was expressed at higher levels in MBI7747 line relative to CCRI45 line during the fiber cell elongation stage. The *GH_D02G0616* gene encoding Polygalacturonase (*At1g48100*) associated with *qFL-D02-1* was upregulated at 5 DPA and 10 DPA in MBI7747 plants relative to levels in CCRI45 plants (Figure 5).

## Discussion

Chromosome segment substitution lines have the same or similar genetic backgrounds to those of receptor parental lines, Studies of these CSSLs can thus separate QTLs based on specific qualitative findings, and the effect values associated with individual genotypes can be accurately assessed within the segregation population. Many QTLs within this population have been subjected to fine-mapping, enabling the cloning of potentially relevant genes of interest (Lavaud et al., 2015). Of the 2,292 SSRs screened in the present study, 37 were found to be polymorphic in MBI7747 cotton line when compared to the parental CCRI45, accounting for 1.61% of the total genome. The MBI7747 line, which exhibited significantly increased fiber length and strength in multiple environments relative to the parental CCRI45, was selected from among 332 CSSLs. The phenotypic differences between the two parental lines are presumed to be due to the introgression of chromosome fragments into the CCRI45 genome. To identify the QTLs and introgressed chromosomal fragments associated with fiber quality, we utilized the MBI7747 and CCRI45 lines to generate F_2_ and F_2:3_ populations. Through phenotypic surveys of these isolated populations and associated analyses of polymorphic SSR markers, we were able to identify the genetic regions and QTLs associated with superior MBI7747 line fiber quality.

In total, 18 QTLs were identified in F_2_ and F_2:3_ populations, of which 15 on chromosomes A07, A12, and D02 were located within 7 QTL hot spots. Among these, four stable QTLs (*qFL-A07-1*, *qFL-A12-2*, *qFL-A12-5*, and *qFL-D02-1*) were detectable in more than two environments. These four QTLs had all been confirmed within our earlier-generation interspecific backcross populations (Shi et al., 2019, 2020) and in our secondary segregating populations constructed using CSSLs (Li et al., 2014, 2015; Zhai et al., 2016). *qFL-A07-1* proximal to CGR6381 may be the same QTL associated with *qFL-C7-3* proximal to NAU1085 (adjoining CGR6381 in genetic mapping) that were identified using our earlier-generation interspecific backcross populations and other secondary segregating populations derived from five other CSSLs on the CCRI36 or CCRI45 lines backgrounds (Jamshed et al., 2016; Song et al., 2017; Deng et al., 2019; Shi et al., 2019, 2020), *qFL-A12-2* proximal to CGR5151 and *qFL-A12-5* proximal to HAU0734 may represent the same QTLs associated with *qFL-C12-4* proximal to NAU4889, which adjoins CGR5151 and HAU0734 in genetic mapping analyses, as identified in our earlier-generation interspecific backcross populations on the CCRI45 background (Shi et al., 2020). *qFL-D02-1* was also confirmed in our earlier-generation interspecific backcross populations on the CCRI45 background (Zhai et al., 2016; Shi et al., 2020).

Through an RNA-seq analysis of 10 DPA fibers from the two parental lines, we were able to identify 59 DEGs associated with these four stable QTLs of interest. Through GO analyses, four of these DEGs were identified as being promising candidate genes associated with three of these QTLs, with none having been found to be a suitable candidate gene associated with *qFL-A07-1*. An *EXPA1* gene and two *PDCB3* genes were located within *qFL-A12-5*, while *CSLD3* was associated with *qFL-A12-2*, and *At1g48100* was associated with *qFL-D02-1*. *EXPA1* and the two *PDCB3* genes were highly expressed in CCRI45 and MBI7747 samples during the rapid elongation stage at 5 and 10 DPA, with such expression being more pronounced in MBI7747 line relative to the parental lines. Prior analyses of Upland cotton have demonstrated that *EXPA1* overexpression can increase fiber length and cotton boll production (Li et al., 2016; Huang et al., 2021). Moreover, *G. hirsutum EXPA1* expression levels have been shown to be under the control of *GhHOX3*, which is a transcription factor that promotes the elongation of cotton fibers (Shan et al., 2014), thus further supporting a likely role for *EXPA1* as a mediator of fiber elongation in the MBI7747 sample. In prior reports, cotton fiber elongation has also been shown to be driven by callose temporal accumulation at plasmodesmata (Ruan et al., 2004). The overexpression of *PDCB* genes in Arabidopsis plants has also been linked to augmented plasmodesmata callose deposition (Simpson et al., 2009), thus supporting a potential role for *PDCB3* as a regulator of cotton fiber elongation. The *CSLD3* gene was more highly expressed in MBI7747 samples relative to CCRI45 samples during the early fiber elongation stage from 5 to 25 DPA. CSLD3 has been linked to root hair elongation in Arabidopsis, with the loss of this gene markedly impairing such elongation and resulting in cytoplasmic leakage (Wang et al., 2001). Additionally, the *CSLD3* active site was responsible for the rescue of a cellulose deficiency phenotype (O’Leary 2020). As such, the protein encoded by *CSLD3* may regulate cotton fiber elongation at least in part by influencing primary cell wall synthesis within the root hair tip-growing zone. *PGX3* was highly expressed in both MBI7747 and CCRI45 samples during the rapid elongation stage from 5 to 10 DPA, with such expression being more pronounced in the former of these two strains. Plant cell separation necessitates the activity of *PGX3* and other endogenous pectinases to mediate pectin degradation (Rui et al., 2017). Comparative proteomic analyses have demonstrated that pectin precursor biosynthesis is integral to the elongation of cotton fibers and Arabidopsis root hairs (Pang et al., 2010). Higher PGX3 levels may thus benefit fiber elongation, although further research will be necessary to confirm the link between these candidate genes and such elongation.

While additional analyses such as CRISPR/Cas9-mediated gene knockout or gene overexpression will be essential to formally confirm the relationship between these four candidate genes and the regulation of fiber length, the present QTL mapping, RNA-seq, and qPCR analyses strongly suggest the potentially important role of these genes as regulators of fiber elongation.

## Data Availability Statement

The original contributions presented in the study are included in the article/Supplementary Material, further inquiries can be directed to the corresponding authors.

## Author Contributions

YY and YS designed the experiments. XX and JF collected the sequences and analyzed the transcriptomic data. QL, YZ, and XX participated in SSR genotyping, QTL mapping, and qRT-PCR and RNA extraction. PL, RP, and JG revised the language and manuscript. QL performed the experiments and wrote the manuscript. All the authors read and approved the final manuscript.

## Conflict of Interest

The authors declare that the research was conducted in the absence of any commercial or financial relationships that could be construed as a potential conflict of interest.

## Publisher’s Note

All claims expressed in this article are solely those of the authors and do not necessarily represent those of their affiliated organizations, or those of the publisher, the editors and the reviewers. Any product that may be evaluated in this article, or claim that may be made by its manufacturer, is not guaranteed or endorsed by the publisher.

## Footnotes

1
https://www.cottongen.org/data/download/marker

2
http://ccb.jhu.edu/software/tophat/index.shtml

3
https://anaconda.org/bioconda/gfold

4
https://www.ncbi.nlm.nih.gov/tools/primer-blast

5
https://blast.ncbi.nlm.nih.gov/
