# Supplementary material for: Identification of Candidate Cotton Genes Associated With Fiber Length Through Quantitative Trait Loci Mapping and RNA-Sequencing Using a Chromosome Segment Substitution Line
Source: Front Plant Sci. 2021 Dec 14;12:796722. doi: 10.3389/fpls.2021.796722 (PMC8712442; doi:10.3389/fpls.2021.796722)
Supplement: Supplementary file 1 [file Table_1.DOC]

Table.S1 Primers used for qRT-PCR in this study.

| Primer Names | Primer Sequences (5′–3′) |
| --- | --- |
| *GH_A12G1972-F* | GCGTGTGGATATGGGAACCT |
| *GH_A12G1972-R* | GCCAGCATTGTTTGGGAGTG |
| *GH_A12G2014-F* | GTTCTTACCCCTCCAGTGCC |
| *GH_A12G2014-R* | GTGCCGACTCCTCCTAACAC |
| *GH_A12G2259-F* | GCACTGTCCCTCTTTTCGGA |
| *GH_A12G2259-R* | TCGATACCCGCAATCACCTT |
| *GH_A12G0616-F* | GGACCTTGTGACAGCCCTAC |
| *GH_A12G0616-R* | TCCTGGACCGATCGAAATGC |
| *β-Actin-F* | ATCCTCCGTCTTGACCTTG |
| *β-Actin-R* | TGTCCGTCAGGCAACTCAT |
